# The Role of Negative Age Stereotypes and Sociodemographic Factors in the Intention to Leave Among German University Hospital Nursing Staff

**DOI:** 10.1177/00469580241277912

**Published:** 2024-09-19

**Authors:** Madeleine Helaß, Anja Greinacher, Andreas Müller, Hans-Christoph Friederich, Imad Maatouk, Christoph Nikendei

**Affiliations:** 1 University Hospital of Würzburg, Würzburg, Bayern, Germany; 2 University of Mannheim, Mannheim, Baden-Württemberg, Germany; 3 University of Duisburg-Essen, Duisburg, Nordrhein-Westfalen, Germany; 4 University Hospital Heidelberg, Heidelberg, Baden-Württemberg, Germany

**Keywords:** nursing staff, age stereotypes, intention to leave, nursing shortage

## Abstract

Staff shortages are a global problem in the nursing profession. Negative beliefs about older workers may have detrimental effects on the development and performance capacity of an aging workforce. To date, little is known about the impact of age stereotypes and potential factors on nurses’ intent to leave (ITL). Therefore, the aim of our study was to assess intention to leave and potential predictors (eg, sociodemographic characteristics and age stereotypes) in a large representative sample of nurses in a German university hospital setting. A total of 423 nurses at the University Hospital of Heidelberg participated in a cross-sectional questionnaire study assessing sociodemographic data, age stereotypes using the “Beliefs About Older Workers” questionnaire, and participants’ intentions to leave and give up their profession. Questionnaires were returned by 423 nurses (13.7% response rate). The results revealed that negative age stereotypes were highly prevalent. Significant correlations between age and negative age stereotypes were found, indicating that the younger the nurses were, the more negative their age stereotypes were. Most nurses with negative age stereotypes had no intention to leave their profession; however, the majority of nurses could not imagine working in the profession until they retired. Despite the low response rate, the results of the current study suggest that organizational and societal measures to reduce age stereotypes should be directed at newcomers and young nurses to retain them in the profession in the long term.

**What we already know about the topic**?
Ageism is related with poorer mental and physical health and could discourage nurses from remaining the job.
**How does the research contribute to this field?**
Negative age stereotypes and age are associated with intention to leave the job and giving up the profession. Age stereotypes and ageism is a problem, especially among young nurses.
**What are the implications toward theory, practice, and policy?**
Future research should focus on longitudinal designs, for example, cohort studies, which proof the associations.

According to the 2020 State of the World Nursing Report, nurses comprise the largest single occupational group among health workers (59% in total).^1^ Unsurprisingly, the current nursing shortage constitutes an increasing global problem^2^ and will determine future world health policy.^3^ The “State of the Worlds Nursing” estimated that by 2030, the health care worker deficit will reach 5.7 million.^1^ The reasons for this development may be constant and consistently high expectations and work demands due to patient-related, professional, environmental, organizational and personal factors.^4^ Brücher and Deufert^5^ examined the German situation and stated that over a 22-year period, significant savings of −29.3% were achieved by decreases in hospital beds (119 000 beds in total), savings of −23% were achieved by decreases in patient care and treatment days (43.1 million patient days in total), and savings of −39% were achieved by decreases in length of hospital stay in days (4.7 days on average). These changes occurred together with a parallel +26.5% increase in the number of treated patients, representing an additional burden of 4.1 million patients.

To compensate for these shortages, job migration, however, is not regarded as an effective countermeasure^6^ since the number of nurses assigned is low. Moreover, the current ongoing demographic changes will induce an additional double-sided problematic effect on this alarming societal development. On the one hand, the number of patients in need of care will continuously increase due to demographic changes.^7^ These patients are characterized by complex somatic multimorbidities and severe psychosocial constraints.^8,9^ On the other hand, aging structures in nursing staff themselves will undergo considerable changes. One in six nurses in the world is aged 55 years or older and is expected to retire in the next 10 years, according to a statistical report.^10^ This may result in a significantly decreased percentage of nurses facing a highly demanding working profile.

Even though more aged nursing staff will be in charge of caring for an increased number of complex multimorbid elderly patients with significant psychosocial constraints, job profile demands are still on the rise. Medical and technical innovations, the digitalization of medicine and increased patient turnover require corresponding professional skills. This may result in a mismatch of needed engagement to cope with these requirements on the one hand and perceived gratification on the other hand.^11^ This phenomenon is well known as the “gratification crisis” model established by Siegrist.^12^ Nursing staff may tend to be even more vulnerable to this condition, as nursing staff often neglect personal needs and are driven by altruistic motives.^13^ Indeed, nursing staff are characterized by high rates of emotional distress and mental disorders^11^ and an increasing number of burnout symptoms during their professional careers^14,15^; the related prevalence rates exceed more than 60% when nurses spend more than 30 years in their job. These observations are consistent with the greater number of sick days and higher hospitalization rates due to mental health problems in nursing staff than in other employees in the general population.^16^ Accordingly, perceived “workability” significantly declines in nursing staff aged older than 45 years^17^ and is associated with an intensified desire and intention to leave (ITL) the job.^18,19^

One important way to address this shortage in nursing is to postpone retirement. However, in most countries, only a small proportion of nurses remain on the job until they reach the official retirement age. Individual factors, such as physical and mental health or financial aspects, as well as work-related factors (eg, changes in work climate or lack of opportunities for development), are important variables in fostering early withdrawal from the nursing profession. In a large European study with more than 28 000 nurses, low levels of self-perceived workability and high levels of psychological strain were strongly associated with an intention to leave the nursing profession.^20^ Furthermore, organizational factors such as insufficient offers for ongoing education or negative age stereotypes toward older workers seem to influence individuals’ ITL.^21^

Age stereotypes are “generalized beliefs about the qualities and characteristics about people of a particular age”.^22^ Stereotyping and discrimination attitudes or behaviors against individuals on the basis of their age is collectively called ageism. Organizational structures and soft factors such as company climate and diversity policies determine the level of ageism in the workplace. Older workers are often confronted with negative age stereotypes such as a lower ability to learn, a reduced potential for development or intensified inflexibility. On a management level, older workers are often considered less productive than their younger counterparts.^23^ In light of these findings, it does not seem surprising that several studies have indicated associations between the presence of negative age stereotypes and poorer mental and physical health.^24^ Thus, in organizations with a work climate permissive of ageism, the aging workforce does not receive the support needed to maintain their ability to work. This negative age stereotype and its impact on the self-perception and behavior of older workers (stereotype threat,^25^) may therefore lead to the fulfilment and reinforcement of negative expectations in a dangerous vicious cycle. In a previous review,^21^ the authors assumed that negative age stereotypes could discourage nurses from remaining in their job. However, numerous studies focusing on nursing staff have been limited due to low participation rates; thus, the findings of these studies are often disparate.

Therefore, the aim of the current study was to assess the relationship between age stereotypes and ITL in a large sample at a hospital offering maximum care. We hypothesize that negative age stereotypes are associated with a higher ITL.

## Methods

### Study Context and Study Design

The present study was designed as a cross-sectional questionnaire survey to measure nurses’ age stereotypes and associated ITL. This study is part of the SEEGEN project funded by the German Federal Ministry of Education and Research (BMBF, 01GL1752D), which aims to assess a multicentred cluster-randomized complex mental health care intervention at different hierarchical and functional levels.^26^ As previously published,^27^ the following criteria also applied to this partial survey: all nursing staff was introduced to the survey in team meetings between April and August 2018. Inclusion criteria were age 18 years or older, signed informed consent, and sufficient German language skills. The STROBE checklist (Strengthening the Reporting of Observational studies in Epidemiology; Appendix) was followed.

### Ethical Considerations

The present study was approved by the ethics committee of the ethics committee of the University of Heidelberg (S-005/2018) on September 3rd, 2018. All participants provided written informed consent to participate and were able to withdraw their participation without any disadvantage. The study was conducted in accordance with the Declaration of Helsinki.^28^

### Participants and Procedure

All the nursing staff working in all 8 departments of the University hospital of Heidelberg were invited to participate in the questionnaire survey. The estimated total number of nurses working in these departments is N = 3090. A total of 536 nurses agreed to participate in the study. Since the focus of the present study was on age stereotypes, only the data of participants who completed the “Beliefs about Older Workers” questionnaire were included in the statistical analysis; thus, N = 423 participants were ultimately included (response rate 13.7%). A total of 81.1% (n = 343) of the respondents were female. Most of the participants (67.8%) were registered nurses, 16.1% were registered nurses with management responsibilities, 11.8% were student nurses, 1.4% were nurses’ auxiliaries, and 2.2% indicated having “another” job position. At the time of the survey, participants had been working in nursing care for an average of 17.8 years (SD = 12.7). A total of 91.7% of the participants reported having open-ended contracts, with an average working time of 35.89 h per week. A total of 14.7% said they had further parallel employment. Table 1 displays the sociodemographic data of the study participants.

**Table 1.** Sociodemographic Data of the Study Participants (N = 423).

Sociodemographic data, N = 423		
	n	%
Gender		
Male	79	18.7
Female	343	81.1
Missing	1	0.2
Age		
18-26	106	25.1
27-34	92	21.7
35-43	61	14.4
44-52	84	19.9
53-60	65	15.4
>60	13	3.4
Missing	2	0.5
Marital status		
Married + living together	175	41.4
Divorced	34	8.0
Single	203	48.0
Widowed	8	1.9
Missing	3	0.7
Job position		
Registered nurse with management responsibilities	68	16.1
Registered nurse	287	67.8
Nurse’s auxiliary	6	1.4
Student nurses	50	11.8
Other	10	2.4
Missing	2	0.5
Type of employment contract		
Open-ended	388	91.7
Limited in time	30	7.1
Missing	4	0.9
Secondary employment		
Yes	62	14.7
No	360	85.1
Missing	2	0.2
	*M*	SD
Activity length in nursing	17.84	12.72
Working hours per week	35.85	9.70

### Survey Instruments

#### Sociodemographic data

The following sociodemographic data were collected: age, sex, marital status, job position, type of employment contract, secondary employment, length of nursing activity, and working hours per week.

#### Beliefs about older workers

This questionnaire surveys attitudes toward older employees. The questionnaire consists of 27 items that are answered on a 5-point Likert scale ranging from 1 (strongly agree) to 5 (strongly disagree). The sum score lies between 27 and 135. The mean scale value of 81 describes a neutral attitude toward older employees. Low values indicate an overall negative attitude toward older employees, while high values indicate an overall positive attitude. The questionnaire has good internal consistency Cronbach’s α = .85^29^; German translations with additional contemporary items show low internal consistencies (.75 - .77), but are still considered sufficient.^30^

#### ITL

We surveyed the intention to leave with 3 questions. Following Camerino et al,^17^ we asked about the intention to terminate the job [ITJ]: “How often in the last 12 months have you thought about leaving your job at this organization?” and giving up the nursing profession (GUP) with “How often in the last 12 months have you thought of giving up your nursing profession and starting another job?” These questions were answered on a 5-point Likert scale for which the response options were “never”, “several times a year”, “several times a month”, “several times a week”, and “every day.” Additionally, participants were asked if they could imagine doing their current job until they reached retirement age (WUR) (“Can you imagine working in your current job until retirement age?”). Participants answered on a 3-point Likert scale for which the response options were “yes”, “to a limited extent”, and “no.”

### Data Analysis

A total of 423 participants were evaluated. All the data were coded and analyzed using SPSS^®^-24 software.^31^ The raw data are displayed as the mean values and standard deviations. Comparisons between the group of study participants and comparative samples were carried out using the Welch test due to lack of homoscedasticity. For within-group comparisons, an analysis of variance (ANOVA) was used to analyze differences in age stereotypes (beliefs about older workers) related to sex, age, job position, and family status (for factor categories, see Table 1). Correlations between age, family status, and ITL were calculated using Pearson’s correlation coefficients. The influence of age stereotypes on ITL was calculated using regression analysis.

## Results

### Beliefs About Older Workers

The participants had a mean score of *M* = 84.7 (SD = 10.3) in the “Beliefs About Older Workers” questionnaire. The scale’s midpoint of 81 was considered neutral. Therefore, the participants reported lightly positive age stereotypes. However, in comparison to a study on age stereotypes conducted among employees of a newspaper company, an electric cooperative, and a state authority, the nurses in general showed significantly more negative age stereotypes *P* < .001.^29^; In our sample, the different age groups of the nurses differed significantly in their age stereotypes (F(5,415) = 21.304, *P* < .001). Participants between 18 and 34 years old scored below the scale midpoint of 81 and therefore reported negative age stereotypes, while participants older than 34 years scored above 81 and therefore reported positive stereotypes (see Table 2). Age stereotypes also differed according to job position (*F*(4,416) = 9.204, *P* < .001). Registered nurses with management responsibilities and registered nurses without management responsibilities (*M* = 87.8, SD = 10.4; *M* = 85.3, SD = 9.9, respectively) had significantly more positive age stereotypes than did student nurses (*M* = 77.1, SD = 9.9). Regarding the gender of the nurses, there was no difference found in regard to age (*P* = .770). However, age stereotypes were found to differ significantly according to family status (*F*(3,416) = 13.3, *P* < .001). Bonferroni post hoc correction showed that single nurses (*M* = 81.7, SD = 10.8) reported fewer positive age stereotypes than did participants who were married or lived with their partner (*M* = 88.0, SD = 9.1; *P* < .001).

**Table 2.** Age Stereotypes According to Participant Age Compared to the Comparative Sample (Welch Test).

Study participants				Comparative sample^a^					
	N	*M*	SD	N	*M*	SD	*t*	*P*	df
Beliefs in older workers according to age									
Overall score	423	84.8	10.3	179	89.9	10.5	−5.484	<.001	330
18-26	106	78.0	10.1						
27-34	92	79.9	10.0						
35-43	61	87.5	10.0						
44-52	84	88.3	74						
53-60	65	90.6	8.3						
>60	13	92.8	7.6						

a Employees of a newspaper company, an electric cooperative, and a state authority.^29^

### Intention to Leave (ITL)

The mean scores of the ITJ and GUP were *M* = 2.1 (SD = 1.1), mean score of WUR was *M* = 2.2 (SD = 0.7). Approximately 2.6% of the participants thought about terminate their job every day (ITJ). A total of 6.9% thought about it a few times a week, 21.7% a few times a month, and 31.2% a few times a year. A total of 36.4% had never thought about termination. A total of 1.2% of the values were missing. According to Camerino et al,^17^ thinking about leaving the job every day, several times a week or months is associated with the a high ITL, while never thinking about leaving the job and thinking about it several times a year can be grouped together as having a low ITL. In our study, 31.2% of the nurses reported a high ITL, whereas 18.9% of the nurses in the NEXT study reported the same. Approximately 3.3% of the participants thought about giving up their job every day. A total of 9.3% thought about it a few times a week, 15.8% a few times a month, and 35.2% a few times a year. A total of 35.5% had never thought about termination. A total of 0.9% of the values were missing. A total of 17.3% of the nurses could imagine doing their current job until they reached retirement age. A total of 43.0% could imagine it only to a limited extent, and 38.5% could not imagine it at all. A total of 0.9% of the values were missing.

Age correlated significantly with ITJ (*r* = −.115, *P* < .05), GUP (*r* = −.210, *P* < .01), and WUR (*r* = −.376, *P* < .01), that means the younger the nurses the higher the ITL. GUP (*F*(3,412) = 4.281, *P* = .005) differed significantly according to family status. Bonferroni post hoc correction showed that single nurses thought about giving up their profession (*M* = 2.3, SD = 1.2) more often than married nurses or nurses who were living with their partner (*M* = 1.9, SD = 0.9; *P* = .004).

### Association of Age Stereotypes With ITL

In three regression models (see Table 3), we included beliefs about older workers, age, and family status as predictors of intention to leave (ITL). For ITJ, the beliefs about older workers were found to be a significant predictor (β = −.155, *P* = .004); the model explained 3.3% of the variance. For GUP, the beliefs about older workers were found to be a significant predictor (β = −.140, *P* = .009); the model explained 6,5% of the variance. For WUR, the beliefs about older workers variable (β = −.162, *P* = .001) and age (β = −.281, *P* ≤ .001) was found to be a significant predictor, the model explained 15.8% of the variance. Thus, negative age stereotypes are rather connected with the thought of changing occupations before reaching the pension stage especially in younger nurses.

**Table 3.** Predictors of ITL.

	ITJ			GUP			WUR		
	*B*	β	*P*	*B*	β	*P*	*B*	β	*P*
Constant	3.420		<.001	3.440		<.001	3.504		<.001
Beliefs about older workers	−.016	−.155	.004	−.015	−.140	.009	−.011	−.162	.001
Age	−.023	−.033	.568	−.089	−.123	.032	−.134	−.281	<.001
Family status	0.026	0.025	0.649	0.072	−0.065	0.221	0.027	0.037	0.469
	*R*² = .033			*R*² = .065			*R*² = .158		
	*F*(3,409) = 4.654			*F*(3,410) = 9.571			*F*(3,410) = 25.583		
	*P* = .003			*P* < .001			*P* < .001		

*Note.* ITL = intention to leave; ITJ = intention to leave the job; GUP = giving up the profession; WUR = working until retirement.

## Discussion

The present study aimed to assess age stereotypes and sociodemographic factors related to the intention to leave (ITJ, GUP, WUR). We found that the examined nurses’ age stereotypes were slightly positive but significantly worse than those in the other professions that have been studied. Age stereotypes were found to be more positive in older nurses. Although the intention to leave the profession was generally low among the participants, most of them could not imagine working in this profession until retirement (especially younger nurses). Age stereotypes and age are significant predictors of giving up the nursing profession.

In our study participants had slightly positive images of older workers. However, these values were significantly lower than those in other previously evaluated professions.^29^ In in-depth interviews with this sample, older nurses were described quite positively as experienced, stress-resistant with good intuition and good patient observation skills. However, older nurses were also described as rigid, inflexible, and less physically resilient.^23^ Age stereotypes are shaped by historical and social circumstances. Specifically, campaigns to rejuvenate the workforce and propose early retirement were launched in the 1980s,^32^ which may have led to the development of corresponding personal and social identities and negative perceptions of an aging workforce. Furthermore, by working with elderly people and sick people, nurses may be subject to a perceptual bias that selectively directs perceptions toward the negative attributes of aging, thus promoting negative age stereotypes.^33^

Second, we found that the older the participants were, the more positive their age images were. With increasing age, the divergence of self-images and how others perceive one becomes more pronounced, and cognitive dissonance develops.^34^ Subsequently, such dissonance is reduced by correcting the external image in the direction of the self-image, which is much more positive. Furthermore, this change can also be explained by the fact that younger study participants count themselves as part of the ingroup of young nurses while older nurses are regarded as an outgroup. An ingroup is characterized by common self-esteem features and differentiates itself from the outgroup, that is, elderly individuals (self-serving bias). Older study participants see themselves within an ingroup of elderly individuals and are thus more likely to perceive self-esteem-serving characteristics.^35^ In order to investigate the nature of age-related stereotypes, a qualitative follow-up study was conducted with the participants of the present study. It was found, among other things, that age stereotypes of young and older nurses differ in terms of which generation was to be labeled as “old” when the prejudice was introduced.^23^

Third, most participants in our study did not intend to leave the nursing profession; however, most participants could not imagine remaining engaged in the nursing profession until retirement age. This corresponds to results from further analyses of the study population.^27^ Younger nurses had a greater intention to leave the profession and could less imagine doing their current job until retirement age than older nurses. Regression analysis indicated that negative age stereotyping is a factor that may influence high ITL in young nurses. Our findings support previous empirical research on organizational and professional turnover.^36^ Shapiro et al.^37^ showed that younger nurses have a greater turnover rate than older nurses. This could be related to the fact that younger people are more flexible and motivated to shape their lives according to their own ideas and have more realistic opportunities to change jobs than their older counterparts. With the introduction of academization in nursing education, younger nurses have the opportunity to further qualify and thus achieve employment in higher positions in the hospital hierarchy. In addition to individual factors, working and organizational conditions such as age-related development opportunities in the workplace,^38^ high work density, shift work, etc., also play a role in the turnover rate. However, the turnover tendency of young nurses does not depend solely on age; it also depends on health impairments such as burnout.^37^ The abovementioned authors described that younger nurses are more frequently confronted with violence in the workplace than are older nurses. Thus, the prevalence rates of burnout is higher in this group than that in other age groups due to their young age, as well as due to their low level of work experience.^39^ In contrast, older generations have greater liabilities and obligations (eg, children or loan payments); thus, a secure working environment is preferred. The need for older workers to stay in a secure job also arises from income inequality and old-age poverty due to the combined socioeconomic inequalities of different European pension systems.^40^

## Limitations and Future Research

We investigated the relationship between intention to leave and age stereotypes, as well as demographic variables, in a large sample of nurses. As this is an exploratory study, no sample size was pre-calculated, so that the results can only be seen as indications of possible correlations. However, with a response rate of approximately 14%, only a fraction of all available nurses participated in this study. In addition, only one university hospital was surveyed. Therefore, the generalizability of the findings can be limited, but gender and age distribution correspond to that of the overall sample of German nursing staff; only the youngest age group was twice as large in our study compared to the overall sample of German nursing staff.^41^ To minimize potential bias, the data were collected across all hospital departments, with similar response rates being present across all units. Future studies using longitudinal designs are necessary to corroborate our results. In future studies, other work-related constructs such as job satisfaction or personal life events should be included in the data analysis in order to increase the knowledge gained about the correlations.

## Conclusion

Even though the response rate is relatively low, the results of the current study show that age stereotypes are a problem, especially among young nurses. As already mentioned by Knight et al.^42^ age-stereotype based interventions including educational and intergenerational contact elements can have a positive impact on self-perception as well as on age images.^43^ The results also suggest that young employees and new entrants to the profession should be focused on as target groups for occupational healthcare measures to help reduce the presence of age stereotypes and keep nurses in the profession in the long term. Similarly, improving the working conditions and atmosphere by establishing measures that aim to create a culture of appreciation and recognition can help reduce the intention to either leave the nursing profession or give it up altogether.

## Supplemental Material

sj-doc-1-inq-10.1177_00469580241277912 – Supplemental material for The Role of Negative Age Stereotypes and Sociodemographic Factors in the Intention to Leave Among German University Hospital Nursing Staff Supplemental material, sj-doc-1-inq-10.1177_00469580241277912 for The Role of Negative Age Stereotypes and Sociodemographic Factors in the Intention to Leave Among German University Hospital Nursing Staff by Madeleine Helaß, Anja Greinacher, Andreas Müller, Hans-Christoph Friederich, Imad Maatouk and Christoph Nikendei in INQUIRY: The Journal of Health Care Organization, Provision, and Financing

sj-docx-2-inq-10.1177_00469580241277912 – Supplemental material for The Role of Negative Age Stereotypes and Sociodemographic Factors in the Intention to Leave Among German University Hospital Nursing Staff Supplemental material, sj-docx-2-inq-10.1177_00469580241277912 for The Role of Negative Age Stereotypes and Sociodemographic Factors in the Intention to Leave Among German University Hospital Nursing Staff by Madeleine Helaß, Anja Greinacher, Andreas Müller, Hans-Christoph Friederich, Imad Maatouk and Christoph Nikendei in INQUIRY: The Journal of Health Care Organization, Provision, and Financing
