# Supplementary material for: The Role of Negative Age Stereotypes and Sociodemographic Factors in the Intention to Leave Among German University Hospital Nursing Staff
Source: Inquiry. 2024 Sep 19;61:00469580241277912. doi: 10.1177/00469580241277912 (PMC11418440; doi:10.1177/00469580241277912)
Supplement: sj-docx-2-inq-10.1177_00469580241277912 – Supplemental material for The Role of Negative Age Stereotypes and Sociodemographic Factors in the Intention to Leave Among German University Hospital Nursing Staff [file sj-docx-2-inq-10.1177_00469580241277912.docx]

| Intention to leave | |
| --- | --- |
| ITJ | How often in the last 12 months have you thought about leaving your job at this organisation? |
| GUP | How often in the last 12 months have you thought of giving up your nursing profession and starting another job? |
| WUR | Can you imagine working in your current job until retirement age? |
|  |  |
| Beliefs about older workers | |
| 1 | Older employees suffer fewer accidents at work. |
| 2 | Most companies are unfair to older workers. |
| 3 | Older workers are more difficult to train for new jobs. |
| 4 | Older workers are absent more often than younger workers. |
| 5 | Younger workers have more serious mishaps than older workers. |
| 6 | If two workers have similar skills, I would choose the older one to work with. |
| 7 | Physical impairments (e.g. back pain, headaches, etc.) are more common among older workers. |
| 8 | The work performance of older employees is of better quality. |
| 9 | Older employees are more grumpy (dissatisfied) at work. |
| 10 | Younger employees are more cooperative at work. |
| 11 | Older employees are more reliable. |
| 12 | Most older employees cannot keep up with the pace of work at work. |
| 13 | Older employees are more loyal to their companies/organisations. |
| 14 | Older workers resist change and are too stuck in their rut. |
| 15 | Younger workers are more interested in challenging work than older workers |
| 16 | Older workers can learn new tasks just as quickly as any other employee |
| 17 | Older workers are the more productive workers |
| 18 | Older employees do not want tasks with increasing responsibility. |
| 19 | Older workers are not interested in learning new skills. |
| 20 | Older workers should step aside (take a less challenging job). |
| 21 | The majority of older workers would stop working if they could afford to. |
| 22 | Older employees are usually sociable and friendly at work. |
| 23 | Compared to when they started their careers, older employees prefer less challenging activities. |
| 24 | It is a better investment to promote younger workers than older workers. |
| 25 | Older workers in our team/work group work just as hard as anyone else. |
| 26 | If I had the choice, I would not want to work with an older worker on a daily basis. |
| 27 | A person's performance decreases sharply with age. |
